# Targeting redox homeostasis in rhabdomyosarcoma cells: GSH-depleting agents enhance auranofin-induced cell death

**DOI:** 10.1038/cddis.2017.412

**Published:** 2017-10-05

**Authors:** Karoline Johanna Habermann, Leon Grünewald, Sjoerd van Wijk, Simone Fulda

**Affiliations:** 1 Institute for Experimental Cancer Research in Pediatrics, Goethe-University, Komturstrasse 3a, Frankfurt, Germany; 2 German Cancer Consortium (DKTK), Partner Site Frankfurt, Germany; 3 German Cancer Research Center (DKFZ), Heidelberg, Germany

## Abstract

Rhabdomyosarcoma (RMS) cells have recently been reported to be sensitive to oxidative stress. Therefore, we investigated whether concomitant inhibition of the two main antioxidant defense pathways, that is, the thioredoxin (TRX) and the glutathione (GSH) systems, presents a new strategy to trigger cell death in RMS. In this study, we discover that GSH-depleting agents, i.e. γ-glutamylcysteine synthetase inhibitor, buthionine sulfoximine (BSO) or the cystine/glutamate antiporter inhibitor erastin (ERA), synergize with thioredoxin reductase (TrxR) inhibitor auranofin (AUR) to induce cell death in RMS cells. Interestingly, AUR causes accumulation of ubiquitinated proteins when combined with BSO or ERA, in line with recent reports showing that AUR inhibits the proteasome besides TrxR. Consistently, AUR/BSO or AUR/ERA cotreatment increases ubiquitination and expression of the short-lived proteins NOXA and MCL-1, accompanied by increased binding of NOXA to MCL-1. Notably, NOXA knockdown significantly rescues RMS cells from AUR/BSO- or AUR/ERA-induced cell death. In addition, AUR acts together with BSO or ERA to stimulate BAX/BAK and caspase activation. Of note, BSO or ERA abolish the AUR-stimulated increase in GSH levels, leading to reduced GSH levels upon cotreatment. Although AUR/BSO or AUR/ERA cotreatment enhances reactive oxygen species (ROS) production, only thiol-containing antioxidants (i.e., *N*-acetylcysteine (NAC), GSH), but not the non-thiol-containing ROS scavenger *α*-Tocopherol consistently suppress AUR/BSO- and AUR/ERA-stimulated cell death in both cell lines. Importantly, re-supply of GSH or its precursor NAC completely prevents AUR/ERA- and AUR/BSO-induced accumulation of ubiquitinated proteins, NOXA upregulation and cell death, indicating that GSH depletion rather than ROS production is critical for AUR/BSO- or AUR/ERA-mediated cell death. Thus, by demonstrating that GSH-depleting agents enhance the antitumor activity of AUR, we highlight new treatment options for RMS by targeting the redox homeostasis.

RMS is the most common soft-tissue sarcoma found in children^1^ and comprises two major histological subtypes, that is, alveolar RMS (ARMS) and embryonal RMS (ERMS).^2, 3, 4^ As the prognosis especially after metastasis or relapse still remains poor,^5^ there is a high medical need for new therapies.

Evasion of programmed cell death belongs to the typical hallmarks of cancer^6^ and can contribute to tumor progression as well as to treatment resistance.^7^ Apoptosis is one of the most extensively studied forms of programmed cell death, consisting of the extrinsic (death receptor) and the intrinsic (mitochondrial) pathway.^8^ Within the latter, pro- and antiapoptotic proteins of the B-cell lymphoma (BCL-2) family are involved in regulating mitochondrial outer membrane permeabilization, resulting in caspase-dependent or -independent cell death.^9, 10, 11^ The relative dominance of proapoptotic compared with antiapoptotic BCL-2 family proteins determines apoptosis sensitivity via homo- or heterodimeric binding. For example, the proapoptotic BCL-2-homology 3 (BH3)-only protein NOXA promotes apoptosis by binding to the antiapoptotic protein MCL-1, thereby antagonizing its antiapoptotic function.^12^ Besides apoptosis, ferroptosis is another form of programmed cell death that has recently been defined.^13^ Ferroptosis depends on iron, as well as on lipid-based reactive oxygen species (ROS) production and lipid peroxidation.^13, 14^

Tight regulation of the redox homeostasis is of vital importance for cells. At low concentrations, ROS exert important functions in many biological and biochemical processes, whereas at high levels ROS can lead to cell death.^15^ Cancer cells often exhibit increased basal ROS levels, for example, because of their elevated metabolic activity, oncogene activation or mitochondrial dysfunction that are compensated by concomitant upregulation of antioxidant pathways to cope with higher levels of oxidative stress.^16^ These changes render cancer cells particularly susceptible to treatment regimens targeting their redox homeostasis.^17^

The glutathione (GSH) and the thioredoxin (TRX) system are two important ROS scavenging pathways that are often upregulated in cancer cells.^18, 19^ As the reduced form of GSH is the most abundant non-protein thiol in the cell,^20^ changes in intracellular GSH levels have an important role in regulating redox homeostasis. As key antioxidant systems can display widespread redundant functions,^21, 22, 23, 24, 25^ simultaneous inhibition of more than one pathway may be required to cause oxidative stress in cancer cells.

Several pharmacological agents can inhibit antioxidant pathways. For example, BSO inhibits γ-glutamylcysteine synthetase,^26^ the rate-limiting enzyme of the GSH synthesis,^27^ leading to GSH depletion.^26, 27^ ERA blocks the cystine/glutamate antiporter at the plasma membrane^13^ that is responsible for the intracellular uptake of cystine, an essential precursor for GSH synthesis.^27^ AUR is a well-known inhibitor of thioredoxin reductase (TrxR),^28^ causing a deficiency of the reduced form of TRX, an important ROS scavenging enzyme.^29^ In addition, AUR has recently been described to block proteasome-associated deubiquitinases (DUBs), thereby inhibiting degradation of ubiquitinated proteins.^30^

Recent genomic analysis of primary RMS samples revealed features of oxidative damage-induced mutations, pointing to increased oxidative stress in RMS.^31^ In addition, RMS have been shown to upregulate *de novo* synthesis of GSH^32^ indicating that RMS cells increase ROS scavenging systems to cope with elevated ROS levels. Furthermore, there is recent evidence showing that RMS cells may be sensitive to ROS-inducing agents.^31^ Against this background, we investigated in this study whether targeting the cellular redox homeostasis represents a suitable approach to induce cell death in RMS.

## Results

### GSH-depleting drugs enhance AUR-induced cell death and suppression of colony formation

To test the hypothesis that concomitant inhibition of the two major antioxidant defense pathways provides a novel strategy to trigger programmed cell death in RMS cells, we blocked in parallel the GSH system by using BSO or ERA and the TRX system by using AUR. The ERMS cell line RD and the ARMS cell line RH30 were used as cellular models to represent the two major histopathological subtypes of RMS.

**Figure 1:**
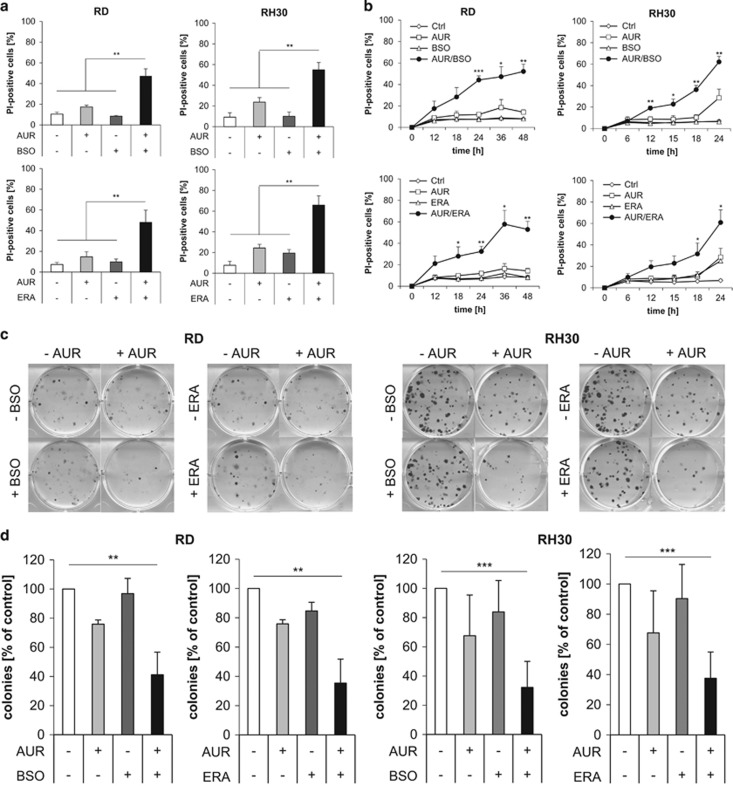
GSH-depleting drugs enhance AUR-induced cell death and suppression of colony formation. (**a**) RMS cells were treated for 24 h (RH30) or 48 h (RD) with 1 *μ*M AUR and/or 1 *μ*M BSO and/or ERA (RH30: 1 *μ*M, RD: 2 *μ*M). Cell death was determined by PI staining using flow cytometry. Mean and S.D. of at least three independent experiments carried out in triplicate are shown; **P≤0.01. (**b**) RMS cells were treated with 1 *μ*M AUR and/or 1 *μ*M BSO and/or ERA (RH30: 1 *μ*M, RD: 2 *μ*M) for indicated times. Cell death was determined by PI staining using flow cytometry. Mean and S.D. of at least three independent experiments carried out in triplicate are shown; *P≤0.05, **P≤0.01, ***P≤0.001 (**c** and **d**) Cells were treated with 1 *μ*M AUR and/or 1 *μ*M BSO and/or ERA (RH30: 1 *μ*M, RD: 2 *μ*M) and colony formation was assessed after 10–12 days as described in the Materials and methods section. The number of colonies is expressed as percentage of untreated controls (**d**) and representative images are shown (**c**). Mean and S.D. of at least three independent experiments carried out in triplicate are shown; **P≤0.01, ***P≤0.001

Of note, AUR cooperated with BSO or ERA to significantly increase cell death compared with treatment with either agent alone in both RMS cell lines (Figure 1a). Calculation of combination indices (CIs) showed that the interaction of AUR with BSO or ERA was synergistic (Supplementary Figure 1,Supplementary Tab. 1). Kinetic analysis demonstrated a time-dependent induction of cell death by AUR together with BSO or ERA (Figure 1b).

To explore whether the combination treatments also have an impact on long-term clonogenic survival, we performed colony assays. AUR/BSO cotreatment, as well as AUR/ERA cotreatment significantly diminished the number of colonies compared with untreated controls (Figures 1c and d). These findings demonstrate that GSH-depleting drugs enhance AUR-induced cell death and suppression of colony formation in RMS cells.

### AUR/BSO or AUR/ERA cotreatment triggers ROS production

**Figure 2:**
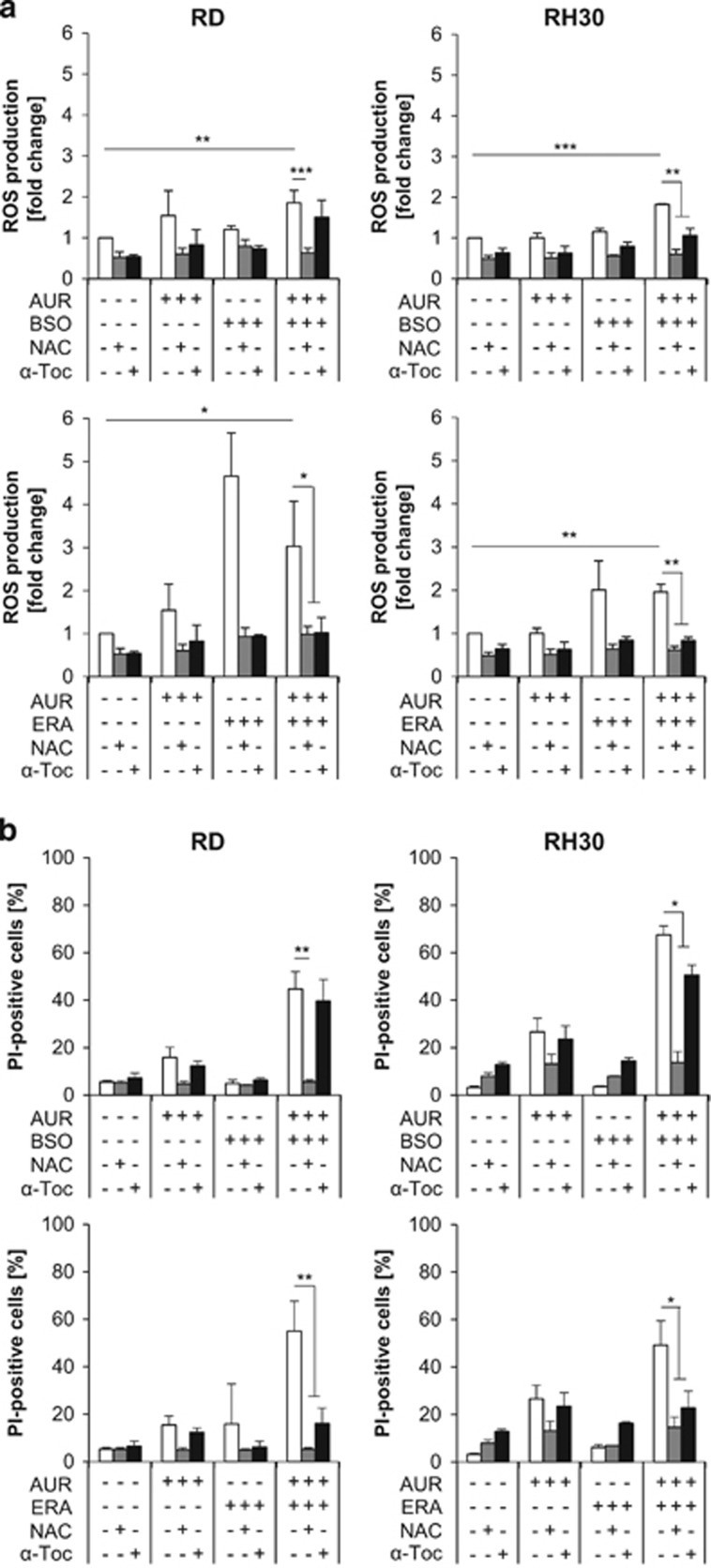
AUR/BSO or AUR/ERA cotreatment triggers ROS production. (**a**) RMS cells were treated for 15 h (RH30) and 18 h (RD) with 1 *μ*M AUR and/or 1 *μ*M BSO and/or ERA (RH30: 1 *μ*M, RD: 2 *μ*M) in the presence and absence of 10 mM NAC or 100 *μ*M *α*-Toc, which were added 1 h before treatment. ROS production was determined by FACS analysis of the viable cell population using the fluorescent dye CM-H2DCFDA and is shown as *x*-fold ROS production compared with control. Mean and S.D. of at least three independent experiments carried out in triplicate are shown; *P≤0.05, **P≤0.01, ***P≤0.001. (**b**) RMS cells were treated for 24 h (RH30) or 48 h (RD) with 1 *μ*M AUR and/or 1 *μ*M BSO and/or ERA (RH30: 1 *μ*M, RD: 2 *μ*M) in the presence and absence of 10 mM NAC or 100 *μ*M *α*-Toc, which were added 1 h before treatment. Cell death was determined by PI staining using flow cytometry. Mean and S.D. of at least three independent experiments carried out in triplicate are shown; *P≤0.05, **P≤0.01

To unravel the underlying mechanisms of synergistic cell death, we determined ROS production. AUR/BSO or AUR/ERA cotreatment significantly increased ROS production in comparison with untreated controls (Figure 2a). To investigate the requirement of ROS for cell death, we used ROS scavengers. Interestingly, the thiol-containing antioxidant and GSH precursor *N*-acetylcysteine (NAC) profoundly suppressed AUR/BSO- and AUR/ERA-stimulated ROS production, as well as cell death (Figures 2a and b). In contrast, the non-thiol-containing ROS scavenger *α*-Tocopherol (*α*-Toc) only partially rescued RH30, but not RD cells from AUR/BSO-induced ROS production and cell death, whereas it protected both RMS cell lines from AUR/ERA-induced ROS production and cell death (Figures 2a and b). These findings suggest that ROS do contribute but do not solely account for the combination treatment-induced cell death.

### AUR/BSO or AUR/ERA cotreatment causes proteasome inhibition and increases ubiquitination and expression of NOXA and MCL-1

**Figure 3:**
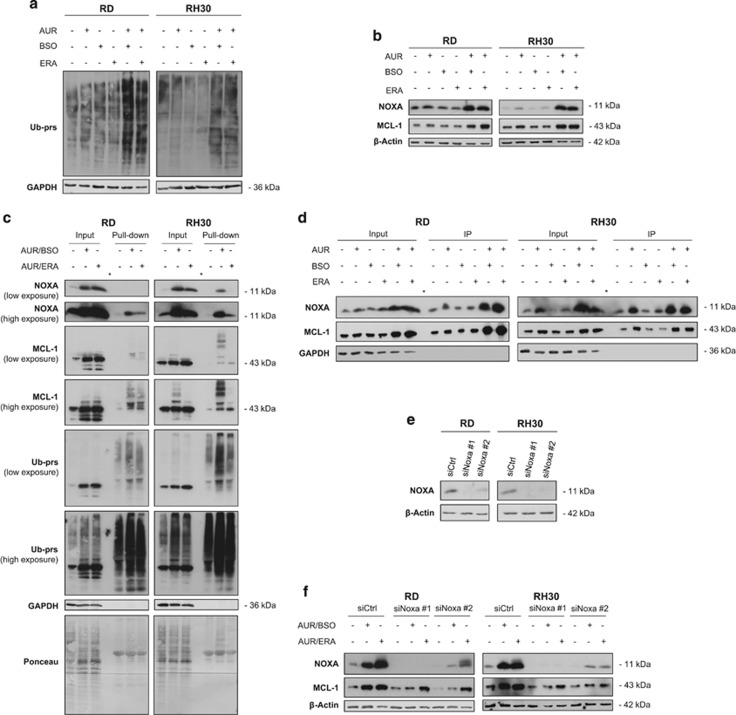
AUR/BSO or AUR/ERA cotreatment causes proteasome inhibition and increases ubiquitination and expression of NOXA and MCL-1. (**a**) RMS cells were treated for 18 h (RH30) and 24 h (RD) with 1 *μ*M AUR and/or 1 *μ*M BSO and/or ERA (RH30: 1 *μ*M, RD: 2 *μ*M). The amount of ubiquitinated proteins (Ub-prs) was assessed by Western blotting, GAPDH served as loading control. (**b**) RMS cells were treated for 18 h (RH30) and 24 h (RD) with 1 *μ*M AUR and/or 1 *μ*M BSO and/or ERA (RH30: 1 *μ*M, RD: 2 *μ*M). Protein expression of NOXA and MCL-1 was assessed by Western blotting, *β*-actin served as loading control. (**c**) Cells were treated for 18 h (RH30) and 24 h (RD) with 1 *μ*M AUR and 1 *μ*M BSO or ERA (RH30: 1 *μ*M, RD: 2 *μ*M). Ubiquitination of MCL-1 and NOXA was assessed by pull-down assay using TUBE-GST-linked agarose beads. The precipitate was analyzed for Ub-prs, NOXA and MCL-1 expression by Western blotting, GAPDH and Ponceau staining served as loading control; asterisks indicate empty lane. (**d**) Cells were treated for 18 h (RH30) and 24 h (RD) with 1 *μ*M AUR and/or 1 *μ*M BSO and/or ERA (RH30: 1 *μ*M, RD: 2 *μ*M). MCL-1 was immunoprecipitated (IP). The precipitate was analyzed for MCL-1 and NOXA expression by Western blotting. GAPDH served as loading control; asterisks indicate empty lane. (**e** and **f**) RMS cells were transiently transfected with siRNA against NOXA or non-targeting control siRNA. (**e**) Protein expression of NOXA was assessed by Western blotting 48 h after knockdown. *β*-Actin served as loading control. (**f**) Cells were treated for 18 h (RH30) and 24 h (RD) after knockdown for 48 h with 1 *μ*M AUR and 1 *μ*M BSO or ERA (RH30: 1 *μ*M, RD: 2 *μ*M). Protein expression of NOXA and MCL-1 was assessed by Western blotting. *β*-Actin served as loading control

As AUR has recently been reported to repress proteasome-associated DUBs apart from inhibiting TrxR,^28, 30^ we next asked whether AUR affects proteasome activity at the concentrations used in our study. To address this question, we assessed accumulation of total ubiquitinated proteins as a marker of proteasome inhibition by Western blot analysis using an antibody against ubiquitinated proteins. Interestingly, cotreatment with AUR/BSO or AUR/ERA caused accumulation of ubiquitinated proteins, whereas treatment with AUR alone had little effects (Figure 3a). As inhibition of the proteasome has been shown to result in upregulation of short-lived proteins, we investigated expression levels and the ubiquitination status of NOXA and MCL-1, two proteins of the BCL-2 family with a rapid turnover.^33, 34^ Consistent with the ability of AUR/BSO or AUR/ERA to inhibit the proteasome (Figure 3a), these cotreatments resulted in upregulation of NOXA and MCL-1 protein levels, whereas AUR alone had little effects (Figure 3b). To specifically analyze the ubiquitination status of NOXA and MCL-1, we precipitated ubiquitinated proteins using a tandem ubiquitin-binding entity (TUBE) pull-down assay^35^ and then probed for binding of NOXA and MCL-1 by Western blotting. This revealed that AUR/BSO or AUR/ERA cotreatment enhanced ubiquitination of NOXA and MCL-1 (Figure 3c). Thus, AUR/BSO or AUR/ERA cotreatment causes proteasome inhibition and increases ubiquitination and expression of NOXA and MCL-1.

As NOXA has been reported to bind to MCL-1 thereby antagonizing its antiapoptotic function,^12^ we next assessed the interaction of NOXA and MCL-1. Indeed, co-immunoprecipitation experiments revealed increased binding of NOXA to MCL-1 upon AUR/BSO or AUR/ERA cotreatment (Figure 3d). We then explored whether genetic silencing of NOXA has an impact on MCL-1 levels. Western blot analysis confirmed that two distinct siRNA sequences caused an efficient silencing of NOXA (Figure 3e). Of note, NOXA knockdown attenuated the AUR/BSO- or AUR/ERA-stimulated increase in MCL-1 expression (Figure 3f), indicating that NOXA contributes to MCL-1 accumulation upon these cotreatments.

### NOXA contributes to AUR/BSO- and AUR/ERA-induced cell death

**Figure 4:**
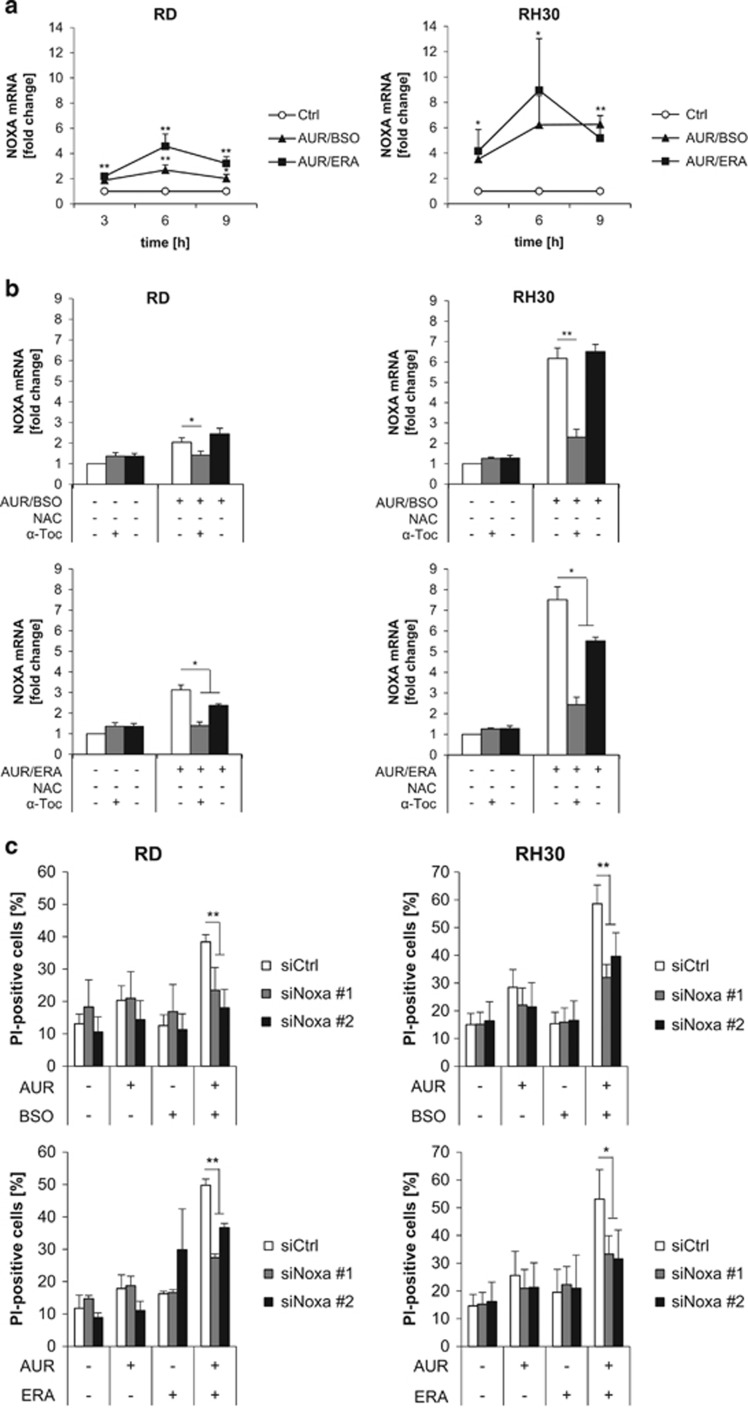
NOXA contributes to AUR/BSO- and AUR/ERA-induced cell death. (**a**) RMS cells were treated for indicated times with 1 *μ*M AUR and 1 *μ*M BSO or ERA (RH30: 1 *μ*M, RD: 2 *μ*M). mRNA expression of NOXA was determined by qRT-PCR, normalized to 28 S expression and is shown as *x*-fold mRNA expression compared with control. Mean and S.D. of at least three independent experiments carried out in duplicate are shown; *P≤0.05, **P≤0.01. (**b**) Cells were treated for 6 h with 1 *μ*M AUR and 1 *μ*M BSO or ERA (RH30: 1 *μ*M, RD: 2 *μ*M) in the presence and absence of 10 mM NAC or 100 *μ*M *α*-Toc, which were added 1 h before treatment. mRNA expression of NOXA was determined by qRT-PCR, normalized to 28S expression and is shown as *x*-fold mRNA expression compared with control. Mean and S.D. of at least three independent experiments carried out in duplicate are shown; *P≤0.05, **P≤0.01. (**c**) RMS cells were transiently transfected with siRNA against NOXA or non-targeting control siRNA and were treated for 24 h (RH30) and 48 h (RD) after knockdown with 1 *μ*M AUR and 1 *μ*M BSO or ERA (RH30: 1 *μ*M, RD: 2 *μ*M). Cell death was determined by PI staining using flow cytometry. Mean and S.D. of at least three independent experiments carried out in triplicate are shown; *P≤0.05, **P≤0.01

To determine whether AUR-based cotreatments also affect mRNA levels of NOXA, we performed quantitative real-time PCR (qRT-PCR) analysis. AUR/BSO or AUR/ERA cotreatment significantly increased NOXA mRNA levels (Figure 4a). Although addition of NAC blocked the AUR/BSO- or AUR/ERA-stimulated increase in NOXA mRNA levels, *α*-Toc partly reduced the increase in NOXA mRNA levels in AUR/ERA-treated, but not in AUR/BSO-treated cells (Figure 4b). These findings indicate that AUR/BSO or AUR/ERA cotreatment leads to increased NOXA mRNA, as well as protein levels.

To explore the functional relevance of NOXA for cell death induction, we knocked down NOXA by RNA interference (RNAi). Importantly, knockdown of NOXA significantly protected RMS cells from both AUR/BSO- and AUR/ERA-induced cell death (Figure 4c). This shows that NOXA contributes to AUR/BSO- and AUR/ERA-induced cell death.

### AUR/BSO or AUR/ERA cotreatment triggers BAX/BAK activation and loss of mitochondrial membrane potential (MMP)

**Figure 5:**
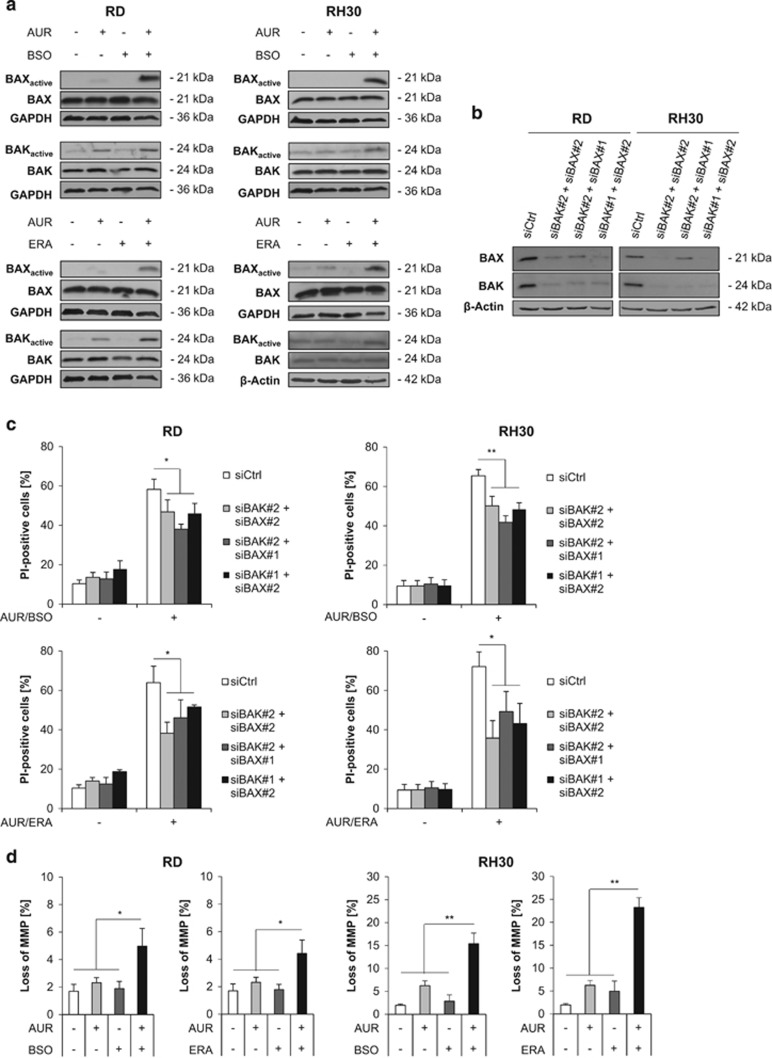
AUR/BSO or AUR/ERA cotreatment triggers BAX/BAK activation and loss of MMP. (**a**) RMS cells were treated for 15 h (RH30) and 18 h (RD) with 1 *μ*M AUR and/or 1 *μ*M BSO and/or ERA (RH30: 1 *μ*M, RD: 2 *μ*M). BAX/BAK activation was assessed by immunoprecipitation using a conformation-specific anti-BAX or anti-BAK antibody and expression of BAX and BAK was analyzed by Western blotting, GAPDH and *β*-actin served as loading control. (**b**) RMS cells were transiently transfected with siRNA against BAX and BAK or non-targeting control siRNA. Protein expression of BAX and BAK was assessed by Western blotting 48 h after knockdown. β-actin served as loading control. (**c**) RMS cells were transiently transfected with siRNA against BAX and BAK or non-targeting control siRNA and were treated for 24 h (RH30) and 48 h (RD) after knockdown with 1 *μ*M AUR and 1 *μ*M BSO or ERA (RH30: 1 *μ*M, RD: 2 *μ*M). Cell death was determined by PI staining using flow cytometry. Mean and S.D. of at least three independent experiments carried out in triplicate are shown; *P≤0.05, **P≤0.01. (**d**) RMS cells were treated for 15 h (RH30) and 18 h (RD) with 1 *μ*M AUR and/or 1 *μ*M BSO and/or ERA (RH30: 1 *μ*M, RD: 2 *μ*M). Loss of MMP was determined by FACS analysis of the viable cell population using the fluorescent dye TMRM. Mean and S.D. of at least three independent experiments carried out in triplicate are shown; *P≤0.05, **P≤0.01

As NOXA is a proapoptotic BH3-only protein known to engage the intrinsic pathway of apoptosis,^36^ we next analyzed activation of BAX and BAK. To this end, we used active conformation-specific antibodies and immunoprecipitation, as BAX/BAK activation is accompanied by conformational changes.^37^ This showed that cotreatment with AUR/BSO or AUR/ERA enhanced activation of BAX and BAK compared with control (Figure 5a). To explore whether BAX/BAK activation is relevant for cell death, we simultaneously knocked down BAX and BAK by siRNAs (Figure 5b). BAX/BAK silencing significantly reduced AUR/BSO-induced as well as AUR/ERA-induced cell death (Figure 5c). In addition, AUR and BSO or ERA cooperated to slightly increase loss of MMP (Figure 5d).

### AUR/BSO- or AUR/ERA-induced cell death is largely caspase-independent

**Figure 6:**
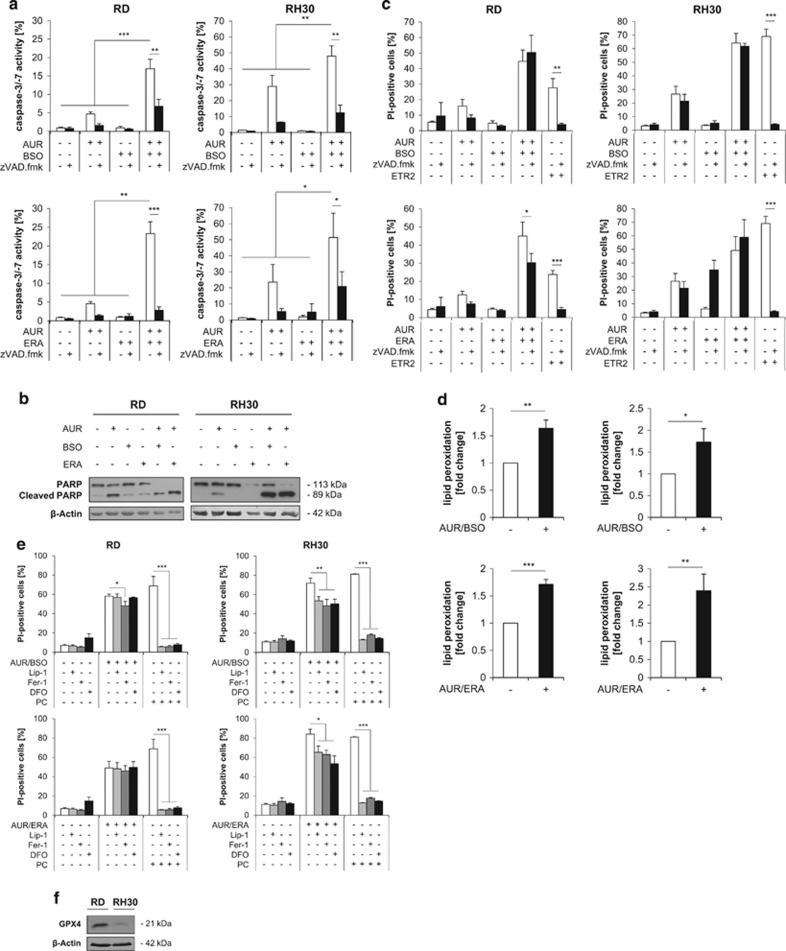
AUR/BSO- or AUR/ERA-induced cell death is largely caspase-independent. (**a**) RMS cells were treated for 24 h (RH30) and 48 h (RD) with 1 *μ*M AUR and/or 1 *μ*M BSO and/or ERA (RH30: 1 *μ*M, RD: 2 *μ*M). Activation of caspase-3 or -7 was detected by fluorescence microscopy using CellEvent Caspase-3/7 Green Detection Reagent. Staining of the cells with Hoechst served as cell count control. Mean and S.D. of at least three experiments performed in triplicate are shown; *P≤0.05, **P≤0.01, ***P≤0.001. (**b**) Cells were treated for 24 h (RH30) and 48 h (RD) with 1 *μ*M AUR and/or 1 *μ*M BSO and/or ERA (RH30: 1 *μ*M, RD: 2 *μ*M). Protein expression of PARP was assessed by Western blotting. *β*-Actin served as loading control. (**c**) Cells were treated for 24 h (RH30) and 48 h (RD) with 1 *μ*M AUR and/or 1 *μ*M BSO and/or ERA (RH30: 1 *μ*M, RD: 2 *μ*M) in the presence and absence of 50 *μ*M zVAD.fmk, which was added 1 h before treatment. 2 *μ*g/ml TRAIL receptor-2 agonistic antibody ETR2 served as positive control for caspase-dependent cell death. Cell death was determined by PI staining using flow cytometry. Mean and S.D. of at least three independent experiments performed in triplicate are shown; *P≤0.05, **P≤0.01, ***P≤0.001. (**d**) RMS cells were treated for 15 h (RH30) and 18 h (RD) with 1 *μ*M AUR and 1 *μ*M BSO or ERA (RH30: 1 *μ*M, RD: 2 *μ*M). Lipid peroxidation was determined by FACS analysis of the viable cell population using the fluorescent dye BODIPY and is shown as *x*-fold change compared with control. Mean and S.D. of at least three independent experiments carried out in triplicate are shown; *P≤0.05, **P≤0.01, ***P≤0.001 (**e**) RMS cells were treated for 24 h (RH30) or 48 h (RD) with 1 *μ*M AUR and/or 1 *μ*M BSO and/or ERA (RH30: 1 *μ*M, RD: 2 *μ*M) or for 24 h with 3 *μ*M ERA (RH30) or 5 *μ*M ERA (RD), which served as positive control (PC), in the presence and absence of 50 nM Lip-1, 5 *μ*M Fer-1 or 25 *μ*M DFO, which were added 1 h before treatment. Cell death was determined by PI staining using flow cytometry. Mean and S.D. of at least three independent experiments carried out in triplicate are shown; *P≤0.05, **P≤0.01, ***P≤0.001. (**f**) Basal protein expression of GPX4 was assessed by Western blotting. *β*-Actin served as loading control

In addition, AUR/BSO and AUR/ERA combination treatment enhanced caspase-3/-7 activity (Figure 6a) and cleavage of poly (ADP-ribose) polymerase (PARP) (Figure 6b), a typical caspase-3 substrate.^38^ Although the addition of the broad-range caspase inhibitor zVAD.fmk significantly reduced AUR/BSO- or AUR/ERA-stimulated caspase-3/-7 activity (Figure 6a), it did not prevent the induction of cell death (Figure 6c). Tumor necrosis factor (TNF)-related apoptosis-inducing ligand (TRAIL) receptor-2 agonistic antibody ETR2 was used as positive control, as it is a prototypic stimulus of caspase-dependent cell death in RMS.^39^ These findings show that AUR/BSO and AUR/ERA cotreatment triggers typical parameters of intrinsic apoptosis, although cell death can proceed in a caspase-independent manner when caspases are blocked.

As ERA and BSO have been reported to induce ferroptotic cell death,^4, 13^ we investigated whether AUR/BSO- or AUR/ERA-induced cell death exhibits ferroptotic features like lipid peroxidation. Indeed, AUR/BSO, as well as AUR/ERA cotreatment significantly increased lipid peroxidation (Figure 6d), although this increase was rather minor compared with RSL3 as a positive control (Supplementary Figure 2). Furthermore, addition of ferroptosis inhibitors including Liproxstatin-1 (Lip-1), Ferrostatin-1 (Fer-1) or the iron-chelating compound deferoxamine (DFO) partially reduced AUR/BSO- and AUR/ERA-induced cell death in RH30 cells, while they had little or no protective effects in RD cells, but completely suppressed ferroptosis induced by RSL3 as positive control (Figure 6e). Consistently, RH30 cells were found to harbor lower constitutive protein expression of GPX4 (Figure 6f), which reduces oxidized lipid hydroperoxides within biological membranes.^40^ Together, these results indicate that ferroptotic signaling pathways partially contribute to AUR/BSO- and AUR/ERA-induced cell death in RH30 cells.

### BSO or ERA counteract the AUR-stimulated increase in GSH levels

**Figure 7:**
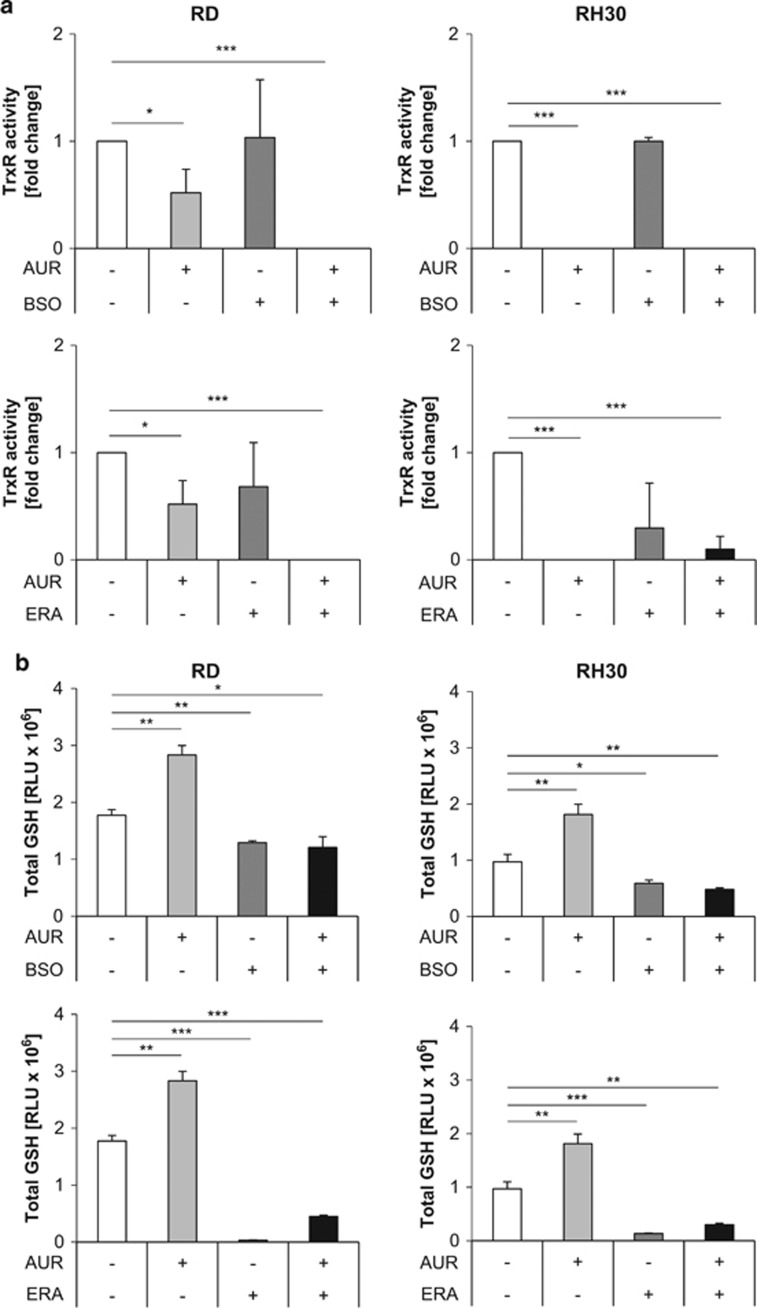
BSO or ERA counteract the AUR-stimulated increase in GSH levels. (**a** and **b**) RMS cells were treated for 15 h (RH30) and 18 h (RD) with 1 *μ*M AUR and/or 1 *μ*M BSO and/or ERA (RH30: 1 *μ*M, RD: 2 *μ*M). (**a**) TrxR activity was determined using the TrxR assay kit colorimetric from Abcam following the instructor's manual. In all, 80 *μ*g of protein were used for each analysis. Activity is shown as *x*-fold expression compared with control. Mean and S.D. of three independent experiments performed in triplicate are shown; *P≤0.05, ***P≤0.001. (**b**) The intracellular amount of total GSH was determined using the GSH/GSSG-Glo Assay from Promega following the manufacturer's information. Luciferase signal (RLU) was normalized to 10 000 cells. Mean and S.D. of three independent experiments performed in triplicate are shown; *P≤0.05, **P≤0.01, ***P≤0.001

To confirm that AUR inhibits TrxR, we assessed TrxR activity. Treatment with AUR alone or in combination with BSO or ERA significantly decreased TrxR activity (Figure 7a). As both BSO and ERA are known to cause GSH depletion,^13, 41^ we next monitored cellular GSH levels. As expected, BSO or ERA alone significantly reduced GSH levels (Figure 7b). Of note, treatment with AUR alone increased GSH levels (Figure 7b), in line with the described compensatory upregulation of the GSH synthesis pathway upon TrxR deficiency.^22^ Importantly, the addition of BSO or ERA abolished this AUR-stimulated increase in GSH levels, leading to reduced GSH levels in cotreated cells (Figure 7b). This shows that BSO or ERA counteract the AUR-stimulated increase in GSH levels.

### Re-supply of GSH rescues AUR/BSO- or AUR/ERA-induced proteasome inhibition, NOXA accumulation and cell death

**Figure 8:**
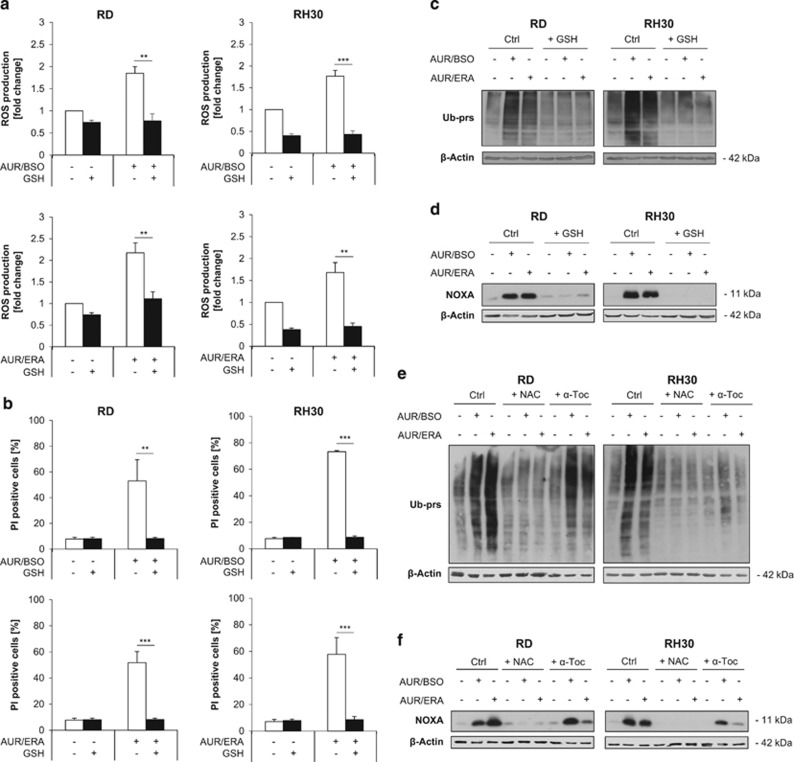
Re-supply of GSH rescues AUR/BSO- or AUR/ERA-induced proteasome inhibition, NOXA accumulation and cell death. (**a**) RMS cells were treated for 15 h (RH30) and 18 h (RD) with 1 *μ*M AUR and 1 *μ*M BSO or ERA (RH30: 1 *μ*M, RD: 2 *μ*M) in the presence and absence of 2.5 mM reduced GSH, which was added 1 h before treatment. ROS production was determined by FACS analysis of the viable cell population using the fluorescent dye CM-H2DCFDA and is shown as *x*-fold ROS production compared with control. Mean and S.D. of at least three independent experiments performed in triplicate are shown; **P≤0.01, ***P≤0.001. (**b**) RMS cells were treated for 24 h (RH30) and 48 h (RD) with 1 *μ*M AUR and 1 *μ*M BSO or ERA (RH30: 1 *μ*M, RD: 2 *μ*M) in the presence and absence of 2.5 mM reduced GSH, which was added 1 h before treatment. Cell death was determined by PI staining using flow cytometry. Mean and S.D. of at least three independent experiments are shown; **P≤0.01, ***P≤0.001. (**c** and **d**) Cells were treated for 18 h (RH30) and 24 h (RD) with 1 *μ*M AUR and 1 *μ*M BSO or ERA (RH30: 1 *μ*M, RD: 2 *μ*M) in the presence and absence of 2.5 mM reduced GSH, which was added 1 h before treatment. Protein expression of ubiquitinated proteins (Ub-prs) and NOXA were determined through Western blotting. *β*-Actin served as loading control. (**e** and **f**) Cells were treated for 18 h (RH30) and 24 h (RD) with 1 *μ*M AUR and 1 *μ*M BSO or ERA (RH30: 1 *μ*M, RD: 2 *μ*M) in the presence and absence of 10 mM NAC or 100 *μ*M *α*-Toc, which were added 1 h before treatment. Protein expression of ubiquitinated proteins (Ub-prs) and NOXA were determined through Western blotting. *β*-Actin served as loading control

The observed decrease of GSH levels in AUR/BSO- or AUR/ERA-cotreated cells may increase AUR's cytotoxicity, as thiol groups have been described to block the activity of AUR.^42, 43^ To further investigate the relevance of the cellular GSH pool in regulating AUR's cytotoxicity, we tested GSH as thiol donor. Importantly, the addition of GSH completely abolished both AUR/BSO- and AUR/ERA-induced ROS production and cell death (Figures 8a and b). Of note, GSH prevented accumulation of ubiquitinated proteins, as well as accumulation of NOXA upon AUR/BSO or AUR/ERA cotreatment (Figures 8c and d). Similar to GSH, addition of NAC, a precursor of GSH and thiol-containing antioxidant, abolished both AUR/BSO- and AUR/ERA-stimulated accumulation of ubiquitinated proteins and NOXA (Figures 8e and f). In contrast, the non-thiol-containing antioxidant *α*-Toc reduced the accumulation of ubiquitinated proteins and NOXA in both AUR/ERA-treated cell lines and in AUR/BSO-treated RH30 cells, but not in AUR/BSO-treated RD cells (Figures 8e and f). These findings are in line with the differential ability of NAC and *α*-Toc to protect RMS cells from AUR/ERA- or AUR/BSO-induced cell death (Figure 2b). Together this set of experiments shows that re-supply of GSH rescues AUR/BSO- or AUR/ERA-induced proteasome inhibition, NOXA accumulation and cell death. This underscores that depletion of cellular GSH levels has an important role in unleashing AUR-induced cell death.

## Discussion

As oxidative stress has recently been identified as a pathway of therapeutic relevance in RMS,^31^ we tested in this study whether concomitant pharmacological inhibition of the two main antioxidant pathways, that is, the TRX and the GSH synthesis pathway, may constitute a new treatment strategy for RMS. Here, we discovered that GSH-depleting agents, that is, BSO or ERA, synergize with AUR to induce cell death in RMS cells. This synergism is confirmed by calculation of CI. The potency of this combination is emphasized by data showing that AUR/BSO or AUR/ERA cotreatment also suppresses long-term clonogenic survival.

**Figure 9:**
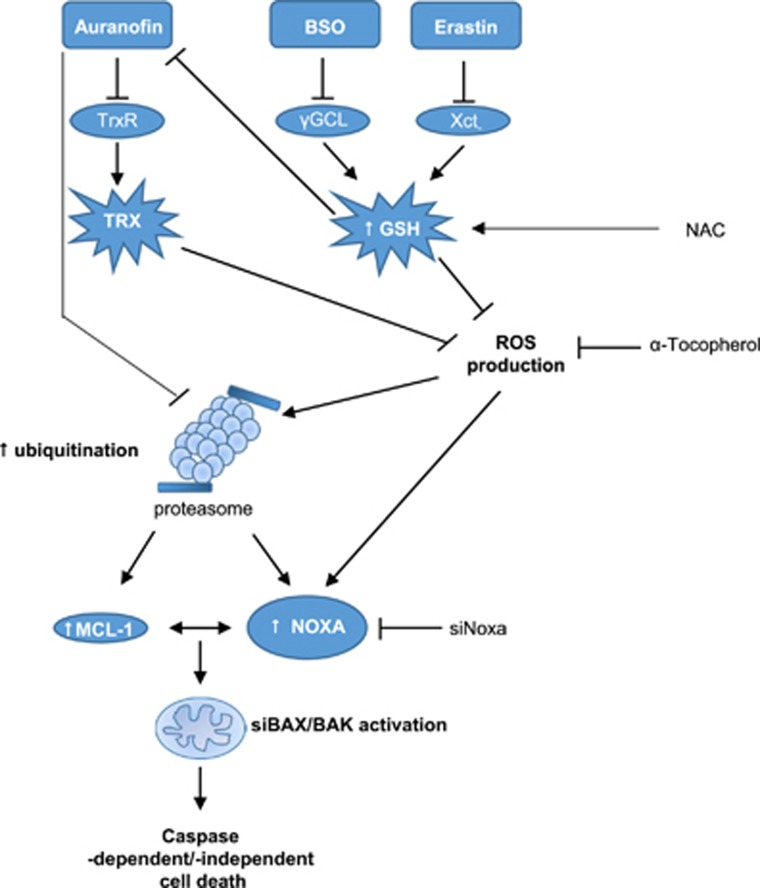
Scheme of AUR/BSO- or AUR/ERA-triggered synergistic cell death. AUR inhibits TrxR, which leads to upregulation of GSH, inhibiting AUR. ERA or BSO cause depletion of GSH levels, thereby counteracting the AUR-mediated upregulation of GSH and increasing AUR's cytotoxic activity. This leads to proteasome inhibition and subsequently to accumulation of ubiquitinated NOXA and MCL-1, followed by activation of BAX/BAK and caspases. See text for more details

Importantly, we identify the BSO- or ERA-stimulated depletion of the cellular GSH pool rather than changes in ROS levels as a relevant mechanism sensitizing cells for AUR-induced cell death (Figure 9). Several lines of evidence support this conclusion. First, BSO or ERA counteract the AUR-stimulated upregulation of GSH levels, resulting in GSH depletion. The increase in GSH levels by AUR points to a compensatory upregulation of the GSH synthesis pathway upon inhibition of the TRX system, in line with the reported redundancy between the GSH and the TRX systems.^21, 22, 23, 24, 25^ Second, replenishing the cellular GSH pool by adding GSH or NAC, a precursor of GSH, completely blocks AUR/BSO- or AUR/ERA-induced cell death. Third, only thiol-containing antioxidants, but not ROS scavengers without thiol groups consistently abrogate AUR/BSO- or AUR/ERA-induced cell death in both RMS cell lines. Changes in intracellular GSH levels likely affect AUR's cytotoxicity, as thiol groups have been described to inactivate AUR via reaction with its gold ion.^42, 43^ This implies that GSH depletion by BSO or ERA can unleash the cytotoxicity of AUR.

Of note, in line with the recently described ability of AUR to inhibit proteasome-associated DUBs,^30^ we provide evidence showing that proteasome inhibition is important for AUR/BSO- or AUR/ERA-induced cell death. This conclusion is supported by our data showing that AUR/BSO or AUR/ERA cotreatment leads to proteasome inhibition, as indicated by the accumulation of ubiquitinated proteins and by enhanced ubiquitination of short-lived proteins such as NOXA and MCL-1. Also, the addition of thiol-containing compounds that block AUR/BSO- or AUR/ERA-induced cell death abolishes proteasome inhibition by AUR/BSO or AUR/ERA, as well as subsequent upregulation of short-lived proteins such as NOXA.

We identify NOXA as an important mediator of AUR/BSO- or AUR/ERA-induced cell death that accumulates in parallel to AUR/BSO- or AUR/ERA-mediated proteasome inhibition. NOXA is a short-lived proapoptotic protein of the BCL-2 family and a known target of the proteasome.^34^ Knockdown experiments showing that NOXA silencing protects from AUR/BSO or AUR/ERA cotreatment indeed confirm that NOXA contributes to AUR/BSO- or AUR/ERA-induced cell death. Consistently, the increase in NOXA levels resulted in enhanced binding and consequently in neutralization of MCL-1 upon AUR/BSO or AUR/ERA cotreatment. In addition to caspases, also caspase-independent mechanisms are likely involved in mediating AUR/BSO- or AUR/ERA-induced cell death, as the pan-caspase inhibitor zVAD.fmk failed to rescue cell death, as the pan-caspase inhibitor zvad.fmk failed to rescue AUR/BSO- or AUR/ERA-induced cell death despite blocking caspase activation. For example, in RH30 cells, ferroptotic signaling pathways may partially contribute to AUR/BSO- and AUR/ERA-induced cell death. Also, we recently identified caspase-independent mechanisms, in this case involving endonuclease G, during vincristine and Polo-like-kinase (PLK) 1 inhibitor-induced apoptosis in RMS cells.^39^

Although we show that proteasome inhibition rather than ROS generation consistently accounts for both AUR/BSO- and AUR/ERA-induced cell death, ROS may contribute to some extent to the induction of cell death, especially in the case of AUR/ERA cotreatment. Upon AUR/ERA cotreatment, ROS production may further enhance proteasome inhibition, as the non-thiol ROS scavenger *α*-Toc reduces AUR/ERA-triggered proteasome inhibition and NOXA accumulation in addition to its protection from cell death. Indeed, ROS have previously been described to inhibit the proteasome.^44, 45^

Our findings have important implications for the development of novel strategies for the treatment of RMS, as AUR or BSO have already been tested in clinical trials, although not in combination. For example, AUR is currently being used in clinical trials for the therapy of recurrent epithelial ovarian, primary peritoneal and fallopian tube cancer^46^ and has been investigated in clinical trials in chronic lymphocytic leukemia.^47^ AUR-based combination therapy that allows dose reduction by exploiting synergistic drug interactions may reduce the risk of side effects that have been described for AUR.^48^ BSO proved to be well tolerated in clinical trials, for example, in combination with melphalan.^49^ The relevance of GSH-depleting strategies for the treatment of RMS is emphasized by a recent report showing that RMS cells harbor elevated GSH levels compared with normal myocytes.^32^ By demonstrating that GSH-depleting agents at subtoxic concentrations synergistically enhance the antitumor activity of AUR, whereas neither GSH depletion nor AUR alone are sufficient to trigger cell death, our study highlights new options targeting cellular redox homeostasis for the treatment of RMS.

## Materials and methods

### Cell culture and chemicals

Human RMS cell lines were obtained from the American Type Culture Collection (Manassas, VA, USA) or from the Deutsche Sammlung von Mikroorganismen und Zellkulturen GmbH (Braunschweig, Germany). Cells were cultured in RPMI-1640 or DMEM GlutaMAX medium (Life Technologies, Inc., Eggenstein, Germany) supplemented with 10% fetal calf serum, 1% penicillin/streptomycin and 1 mM sodium pyruvate (Invitrogen, Karlsruhe, Germany). Chemicals were purchased from Sigma-Aldrich (Steinheim, Germany). Reduced GSH was purchased from Carl Roth (Karlsruhe, Germany). The caspase inhibitor zVAD.fmk was purchased from Bachem (Heidelberg, Germany).

### Determination of cell death and clonogenic growth

Cell death was assessed by analyzing plasma membrane permeability with propidium iodide (PI) staining as described previously using flow cytometry^50^ (FACS Canto II, BD Biosciences, Heidelberg, Germany). To determine colony formation, cells were seeded in a 24-well tissue culture plate, allowed to settle overnight and treated with AUR, BSO, ERA or the combinations for 9 h (RD) or 4 h (RH30). Afterward cells were trypsinized, counted and 200 cells were seeded in a six-well tissue culture plate. Colonies were stained after 10–12 days without medium change with crystal violet solution. Colonies were counted and the percentage of colonies relative to solvent-treated controls was calculated.

### Caspase activity assay

To determine caspase activation, the CellEvent Caspase-3/7 Green Detection Reagent from Thermo Fisher Scientific (Darmstadt, Germany) was used following the instructor's manual. In parallel, cells were stained with 1 *μ*g/*μ*l Hoechst-Dye (Sigma-Aldrich) and measured by fluorescence microscopy (ImageXpress Mikro XLS, Sunnyvale, CA, USA) with automated analysis using MetaXpress Software (Molecular Devices).

### Determination of ROS production, lipid peroxidation, intracellular GSH levels and TrxR activity

To analyze ROS production or lipid peroxidation, medium was discarded, cells were stained for 30 min at 37 °C with 5 *μ*M CM-H_2_DCFDA (Invitrogen) for ROS production or 5 *μ*M BODIPY (Life Technologies, Inc.) for lipid peroxidation. Subsequently, they were trypsinized, centrifuged for 10 min at 4 °C and supernatant was discarded. Cells were resuspended in white RPMI (Life Technologies, Inc.) and immediately analyzed by flow cytometry. To determine GSH levels in the cells the GSH/GSSG-Glo assay (Promega, Madison, WI, USA) was used following the instructor's manual. In parallel, cells were stained with 1 *μ*g/*μ*l Hoechst-Dye (Sigma-Aldrich) and measured by fluorescence microscopy (ImageXpress Mikro XLS) with automated analysis using MetaXpress Software (Molecular Devices). Luciferase signal was normalized to 10 000 cells. TrxR activity was measured with the Thioredoxin Reductase Assay Kit Colorimetric (Abcam, Cambridge, UK) following the instructor's manual. Protein content of the lysates was determined using the Pierce BCA Protein Assay Kit (Thermo Fisher Scientific) and 80 *μ*g of protein were used for each analysis. Negative data appearing after measurement or during analysis were defined as zero, meaning no TrxR activity.

### RNA interference

For transient knockdown by siRNA, cells were reversely transfected with 10 nM or 20 nM SilencerSelect siRNA (Life Technologies, Inc.), that is, control siRNA (4390842 siCtrl) or targeting siRNA (s10709 siNoxa #1, s10710 siNoxa #2) for NOXA; (s1880 siBAK#1, s1881 siBAK#2) for BAK; (s1889 siBAX#1, s1890 siBAX#2) for BAX.

### Western blot analysis

Western blot analysis was performed as described previously,^51^ using the following antibodies: mouse anti-GPX4 (R&D Systems, Wiesbaden, Germany), rabbit anti-NQO1 and mouse IgG1 anti-ubiquitin (P4D1) (Santa Cruz Biotechnology, Santa Cruz, CA, USA), rabbit anti-BAX (Millipore, Darmstadt, Germany), mouse anti-NOXA (Alexis Biochemicals, Grünberg, Germany), rabbit anti-BAK (BD Biosciences), mouse anti-PARP (Cell Signaling, Beverly, MA, USA), rabbit anti-MCL-1 (Stressgen, Victoria, BC, Canada), mouse anti-GAPDH (HyTest, Turku, Finland) or mouse anti-*β*-Actin (Sigma-Aldrich). Goat anti-mouse and goat anti-rabbit with conjugated horseradish peroxidase (Santa Cruz Biotechnology) were used for enhanced chemiluminescence (Amersham Biosciences, Freiburg, Germany) detection or infrared dye-labeled secondary antibodies were used in combination with an infrared imaging system (Odysee Imaging System, LI-COR Biosciences, Bad Homburg, Germany).

### Immunoprecipitation

Immunoprecipitation of active BAX and BAK was performed as previously described.^52^ Briefly, cells were lysed in CHAPS lysis buffer (10 nmol/l HEPES, pH 7.4; 150 nmol/l NaCl; 1% CHAPS). 500–1000 *μ*g protein was immunoprecipitated and incubated overnight at 4 °C with 2 *μ*g/ml mouse anti-BAK antibody (Ab-1; Merck Millipore, Billerica, MA, USA) or anti-BAX antibody (6A7, Sigma-Aldrich) and 10 *μ*l pan-mouse IgG Dynabeads (Life technologies, Inc.), washed with CHAPS lysis buffer and analyzed by western blotting using rabbit anti-BAK antibody (BD Bioscience) or anti-BAX antibody (Merck, Darmstadt, Germany). Immunoprecipitation of MCL-1 was performed in 500 *μ*l lysates containing up to 1000 *μ*g proteins, which were incubated overnight at 4 °C with 2 *μ*g/ml mouse anti-MCL-1 antibody (BD Biosciences) and 10 *μ*l pan-mouse IgG Dynabeads or Protein G Dynabeads (Life Technologies, Inc.) and washed with CHAPS buffer. The precipitate was analyzed for interaction with NOXA by Western blotting using rabbit anti-MCL-1 antibody (ENZO, Lausen, Switzerland) and mouse anti-NOXA antibody (Alexis Biochemicals, Grünberg, Germany).

### TUBE pull-down assay

TUBE pull-down assay was performed as previously described.^35^ Cells were lysed in buffer (50 mM NaCl, 20 mM Tris pH 7,5, 1% NP40, 5 mM EDTA, 10% glycerol) supplemented with a protease inhibitor cocktail tablet (Roche Diagnostics, Mannheim, Germany) for 20 min on ice. In all, 1000 *μ*g of protein were incubated overnight at 4 °C with 50 *μ*l GSH-agarose beads (Sigma-Aldrich) linked to GST-TUBE. Afterward beads were washed with buffer. The precipitate was analyzed for ubiquitin expression by Western blotting using mouse IgG1 anti-ubiquitin (P4D1) (Santa Cruz Biotechnology) antibody and for interaction with NOXA and MCL-1 by Western blotting using rabbit anti-MCL-1 antibody (ENZO), and mouse anti-NOXA antibody (ENZO). To verify the protein amount per lane, the membrane was stained with Ponceau S (AppliChem GmbH, Darmstadt, Germany).

### Determination of MMP

To determine MMP, cells were incubated with TMRM (50 nM; Invitrogen) for 30 min at 37 °C and immediately analyzed by flow cytometry.

### Quantitative real-time PCR

Total RNA was isolated using peqGOLD Total RNA kit from Peqlab Biotechnologie GmbH (Erlangen, Germany) according to the manufacturer's instructions. A total of 1 *μ*g of RNA was used to synthesize the corresponding cDNA using RevertAid H Minus First Strand cDNA Synthesis Kit (MBI Fermentas GmbH, St. Leon-Rot, Germany). To quantify gene expression levels, SYBRGreen-based qRT-PCR was performed using the 7900HT fast real-time PCR system from Applied Biosystems (Darmstadt, Germany). Data were normalized on 28S-rRNA expression as reference gene. Primers are listed in Supplementary Table 2. Melting curves were plotted to verify the specificity of the amplified products. The relative expression of the target gene transcript and reference gene transcript was calculated as ΔΔct. At least three independent experiments in duplicate were performed for each gene.

### Statistical analysis and calculation of the CI values

All results are shown as mean±S.D. Statistical significance was calculated by using Student's *t*-test (two-tailed, two sample, equal variance). CI calculation was performed with CalcuSyn software (Biosoft, Cambridge, UK) with the CI calculation method. Calculated CI values were defined in the following way: CI<0.9 synergistic, 0.9–1.1 additive and CI>1.1 antagonistic. *P*-values were interpreted as follows: *P≤0.05; **P≤0.01; ***P≤0.001.

## Publisher’s Note

Springer Nature remains neutral with regard to jurisdictional claims in published maps and institutional affiliations.
