# Supplementary material for: Targeting redox homeostasis in rhabdomyosarcoma cells: GSH-depleting agents enhance auranofin-induced cell death
Source: Cell Death Dis. 2017 Oct 5;8(10):e3067–. doi: 10.1038/cddis.2017.412 (PMC5680568; doi:10.1038/cddis.2017.412)
Supplement: Supplementary Figures and Tables [file cddis2017412x1.pdf]

Supplementary Legends to Figures and Tables

Suppl. Fig. 1 Combination of AUR with GSH-depleting drugs induces cell death. Cells were treated with the indicated concentrations of AUR, BSO or ERA for 24 hours (RH30) or 48 hours (RD). Cytotoxicity was assessed by plasma membrane permeability using PI staining. Mean and s.d. of at least three experiments performed in triplicate are shown.

Suppl. Fig. 2 ERA at high concentration triggers lipid peroxidation.
RMS cells were treated for 12 hours with 3 µM ERA (RH30) or 5 µM ERA (RD). Lipid peroxidation was determined by FACS analysis of the viable cell population using the fluorescent dye BODIPY and is shown as *x*-fold change compared to control. Mean and s.d. of at least three independent experiments carried out in triplicate are shown; **P* ≤ 0.05, ****P* ≤ 0.001.

Suppl. Tab. 1: CI-values

Suppl. Tab. 2 Primer sequences

Suppl. Figure 1

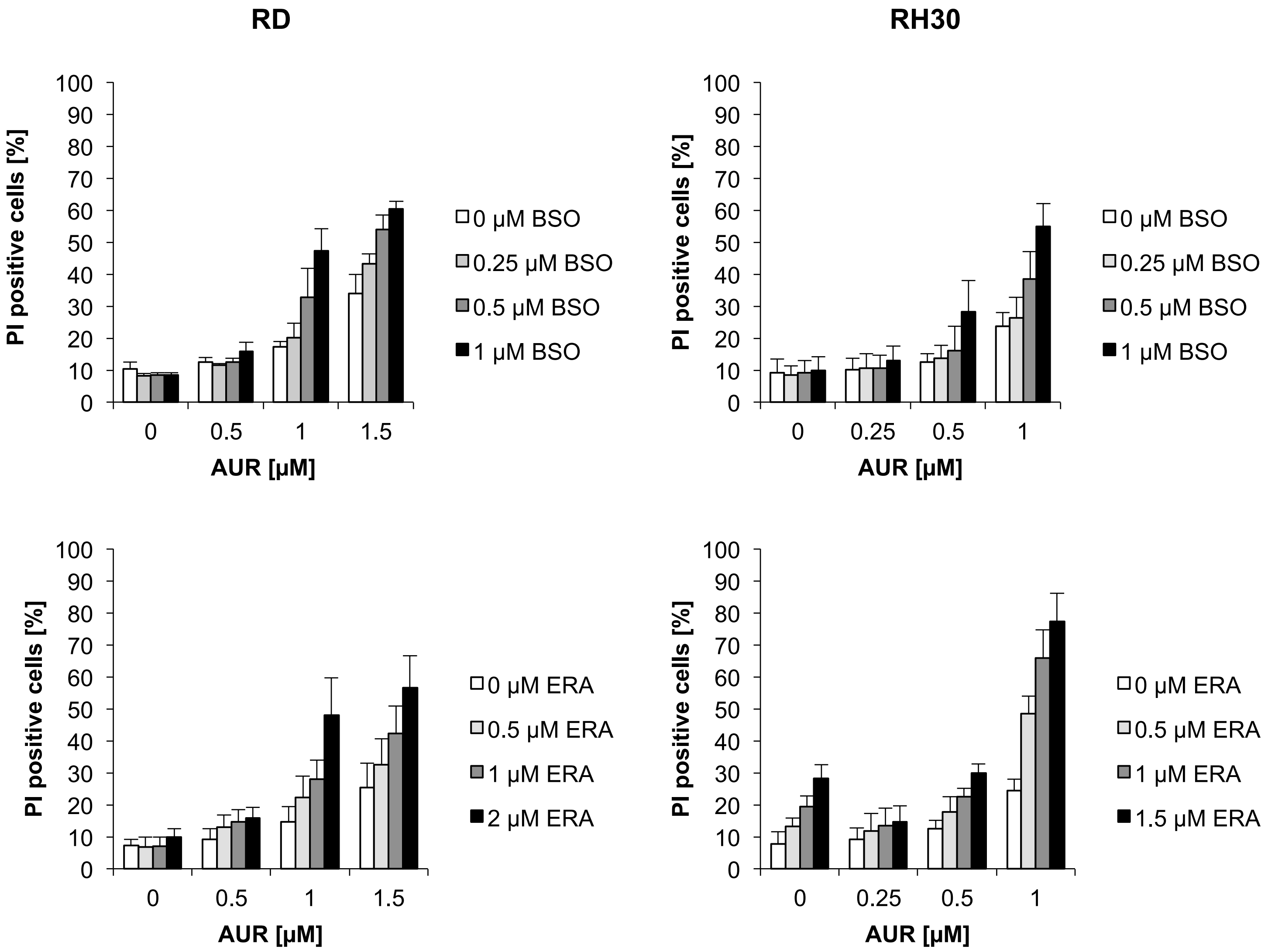

Suppl. Figure 2

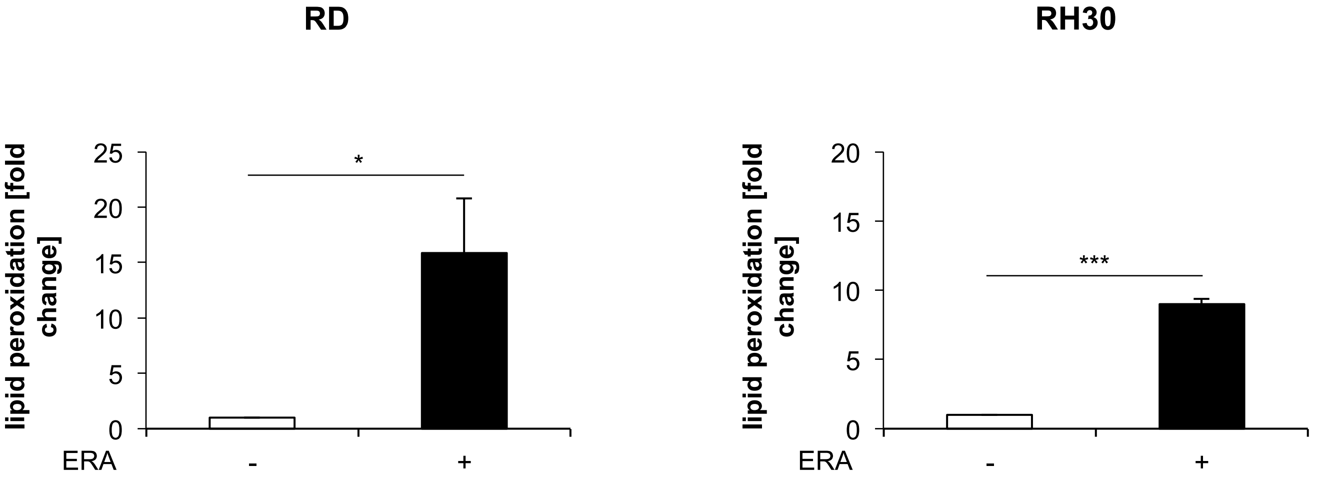

Supplemental Table 1: CI-values

**RD**	**Auranofin [µM]**		
**BSO [µM]**	0.5	1	1.5
0.25	0.985	1.1	**0.603**
0.5	0.92	**0.6**	**0.407**
1	**0.713**	**0.345**	**0.321**

**RH30**	**Auranofin [µM]**		
**BSO [µM]**	0.25	0.5	1
0.25	0.972	1.122	**0.737**
0.5	0.949	**0.86**	**0.345**
1	**0.635**	**0.322**	**0.14**

**RD**	**Auranofin [µM]**		
**Erastin [µM]**	0.5	1	1.5
0.5	**0.728**	**0.724**	**0.665**
1	**0.667**	**0.548**	**0.449**
2	**0.656**	**0.242**	**0.261**

**RH30**	**Auranofin [µM]**		
**Erastin [µM]**	0.25	0.5	1
0.5	1.755	1.334	**0.318**
1	2.389	1.41	**0.19**
1.5	2.99	1.19	**0.128**

Supplemental Table 2: Primer sequences

**Primer**	**Sequence (5'→3')**
h_28S_forward	TTGAAAATCCGGGGGAGAG
h_28S_reverse	ACATTGTTCCAACATGCCAG
h_Noxa_forward	GGAGATGCCTGGGAAGAAG
h_Noxa_reverse	CCTGAGTTGAGTAGCACACTCG
